# Quantification of Immune Variables from Liquid Biopsy in Breast Cancer Patients Links Vδ2^+^ γδ T Cell Alterations with Lymph Node Invasion

**DOI:** 10.3390/cancers13030441

**Published:** 2021-01-25

**Authors:** Stéphane Fattori, Laurent Gorvel, Samuel Granjeaud, Philippe Rochigneux, Marie-Sarah Rouvière, Amira Ben Amara, Nicolas Boucherit, Magali Paul, Marie Mélanie Dauplat, Jeanne Thomassin-Piana, Maria Paciencia-Gros, Morgan Avenin, Jihane Pakradouni, Julien Barrou, Emmanuelle Charafe-Jauffret, Gilles Houvenaeghel, Eric Lambaudie, François Bertucci, Anthony Goncalves, Carole Tarpin, Jacques A. Nunès, Raynier Devillier, Anne-Sophie Chretien, Daniel Olive

**Affiliations:** 1 Team Immunity and Cancer, Centre de Recherche en Cancérologie de Marseille (CRCM), Inserm, U1068, CNRS, UMR7258, Institut Paoli-Calmettes, Aix-Marseille University, UM 105, 13009 Marseille, France; stephane.fattori@inserm.fr (S.F.); laurent.gorvel@inserm.fr (L.G.); ROCHIGNEUXP@ipc.unicancer.fr (P.R.); marie-sarah.rouviere@inserm.fr (M.-S.R.); amira.BEN-AMARA@univ-amu.fr (A.B.A.); nicolas.boucherit@inserm.fr (N.B.); magali.paul@imcheck.fr (M.P.); jacques.nunes@inserm.fr (J.A.N.); DEVILLIERR@ipc.unicancer.fr (R.D.); 2 Cancer Immunomonitoring Platform, Centre de Recherche en Cancérologie de Marseille (CRCM), Inserm, U1068, CNRS, UMR7258, Institut Paoli-Calmettes, 13009 Marseille, France; 3 Systems Biology Platform, Centre de Recherche en Cancérologie de Marseille (CRCM), Inserm, U1068, CNRS, UMR7258, Institut Paoli-Calmettes, Aix-Marseille University, UM 105, 13009 Marseille, France; samuel.granjeaud@inserm.fr; 4 Department of Medical Oncology, Institut Paoli-Calmettes, 13009 Marseille, France; BERTUCCIF@ipc.unicancer.fr (F.B.); GONCALVESA@ipc.unicancer.fr (A.G.); TARPINC@ipc.unicancer.fr (C.T.); 5 Department of Pathology, Institut Paoli-Calmettes, 13009 Marseille, France; DAUPLATM@ipc.unicancer.fr (M.M.D.); THOMASSINJ@ipc.unicancer.fr (J.T.-P.); PACIENCIAM@ipc.unicancer.fr (M.P.-G.); AVENINM@ipc.unicancer.fr (M.A.); JAUFFRETE@ipc.unicancer.fr (E.C.-J.); 6 Department of Clinical Research and Innovations, Institut Paoli-Calmettes, 13009 Marseille, France; PAKRADOUNIJ@ipc.unicancer.fr; 7 Department of Surgical Oncology, Institut Paoli-Calmettes, 13009 Marseille, France; BARROUJ@ipc.unicancer.fr (J.B.); HOUVENAEGHELG@ipc.unicancer.fr (G.H.); LAMBAUDIEE@ipc.unicancer.fr (E.L.); 8 Team Epithelial Stem Cells and Cancer, Centre de Recherche en Cancérologie de Marseille (CRCM), Inserm, U1068, CNRS, UMR7258, Institut Paoli-Calmettes, Aix-Marseille University, UM 105, 13009 Marseille, France; 9 Faculty of Medical and Paramedic Sciences, Aix Marseille University, UM 105, 13005 Marseille, France; 10 Team Predictive Oncology, Centre de Recherche en Cancérologie de Marseille (CRCM), Inserm, U1068, CNRS, UMR7258, Institut Paoli-Calmettes, Aix-Marseille University, UM 105, 13009 Marseille, France; 11 Department of Haematology, Institut Paoli-Calmettes, 13009 Marseille, France

**Keywords:** breast cancers, immune monitoring, liquid biopsy, γδ T cells, mass cytometry, between-group analysis

## Abstract

**Simple Summary:**

Vδ2^+^ γδ T cells have potent antitumor properties both in vitro and in murine preclinical models of breast cancers. However, in the context of human breast cancer, there is a lack of information for potential phenotypic alterations of this crucial immune cell subset. This is partly due to Vδ2^+^ γδ T cells scarcity in surgical specimens. To break this deadlock, we assessed Vδ2^+^ γδ T cell phenotypes using untreated breast cancer patients’ peripheral blood, so-called minimally invasive “liquid biopsy”. We show that circulating Vδ2^+^ γδ T cell phenotypic alterations are already established at diagnosis and related to tumor progression. Notably, terminally differentiated Vδ2^+^ γδ T cells expressing canonical markers of replicative senescence and exhaustion were significantly associated with tumor-draining lymph node invasion. Our results shed light on the interest of using liquid biopsy to monitor rare events and support Vδ2^+^ γδ T cell involvement in breast cancer pathogenesis and progression.

**Abstract:**

The rationale for therapeutic targeting of Vδ2^+^ γδ T cells in breast cancer is strongly supported by in vitro and murine preclinical investigations, characterizing them as potent breast tumor cell killers and source of Th1-related cytokines, backing cytotoxic αβ T cells. Nonetheless, insights regarding Vδ2^+^ γδ T cell phenotypic alterations in human breast cancers are still lacking. This paucity of information is partly due to the challenging scarcity of these cells in surgical specimens. αβ T cell phenotypic alterations occurring in the tumor bed are detectable in the periphery and correlate with adverse clinical outcomes. Thus, we sought to determine through an exploratory study whether Vδ2^+^ γδ T cells phenotypic changes can be detected within breast cancer patients’ peripheral blood, along with association with tumor progression. By using mass cytometry, we quantified 130 immune variables from untreated breast cancer patients’ peripheral blood. Supervised analyses and dimensionality reduction algorithms evidenced circulating Vδ2^+^ γδ T cell phenotypic alterations already established at diagnosis. Foremost, terminally differentiated Vδ2^+^ γδ T cells displaying phenotypes of exhausted senescent T cells associated with lymph node involvement. Thereby, our results support Vδ2^+^ γδ T cells implication in breast cancer pathogenesis and progression, besides shedding light on liquid biopsies to monitor surrogate markers of tumor-infiltrating Vδ2^+^ γδ T cell antitumor activity.

## 1. Introduction

Breast cancer (BC) remains the most diagnosed and leading cause of cancer death among women worldwide [1]. Alongside a major overcoming stalemate is the paucity of information regarding breast tumor’s composition and spatiotemporal dynamics occurring throughout tumor progression [2,3,4]. Notwithstanding, advanced knowledge of the tumor-host immune system interaction allowed an immune-guided BC stratification [5,6,7] and implementation of promising immunotherapeutic clinical trials [8,9]. Indeed, the immune composition of breast cancers has been widely described [10,11], allowing a more comprehensive knowledge of parameters that account for breast tumors’ heterogeneity, progression, and therapeutic targeting. Foremost, combined quantification of tissue-based immune variables such as the density, location, nature, and functional state of tumor-infiltrating lymphocyte (TILs) carry prognostic and predictive values of response to conventional cytotoxic and immunotherapeutic treatments [12,13,14].

However, exploring antitumor immunity patterns in the tumor bed using resected or biopsied primary or metastatic tumors shows multiple constraints. First, apart from being invasive, tumor biopsies are not always repeatedly feasible and are therefore poorly suitable for longitudinal studies or transfer in clinical routine. Second, genetic and immunological heterogeneity exists between and within each metastasis and the primary tumor, which contributes to the complexity of antitumor immunity pattern study using solid biopsies [15,16]. Those limitations have yielded a growing interest in finding minimally invasive methods to assess antitumor immune response. For instance, the quantification of immune variables from liquid biopsy has shown that circulating T cells’ repertoire, proliferation, expansion and immune checkpoint expression correlated with responses to conventional chemotherapy and immune checkpoint inhibitors [17,18,19,20,21,22]. Furthermore, tumor antigen-experienced T cells are present within patients’ peripheral blood [23,24] and circulating cytotoxic T cells, or regulatory T cells (Treg) correspond to those of their counterpart tumor-infiltrating lymphocytes (TILs) [25,26]. In addition, because only small fragments of the surgical specimen are usually available for immunomonitoring studies, investigations based on tumor biopsies may be biased toward the predominantly represented immune subtypes, thus limiting the characterization of rare immune populations in breast tissues. Thus, phenotyping of peripheral blood mononuclear cells (PBMCs) may shed light on rare immune populations such as γδ T cells that yet impact the clinical outcome of patients with malignancies [27,28].

Human γδ T cells represent a unique conserved lineage of T cells, which are responsive against viral/microbial pathogens and transformed cells [29]. Similar to conventional αβ CD8^+^ T cells, γδ T cells display pro-inflammatory cytokine production and cytotoxic effector function. As for αβ CD8^+^ T cells, γδ T cells can be divided into four functionally distinct subsets reflecting their maturation stages, using a combination of canonical markers known to be differentially expressed during the course of T cell maturation. That is, following antigenic stimulation, CD45RA^+^ CCR7^+^ CD27^+^ CD28^+^ naïve γδ T cells mature to CD45RA^−^ CCR7^+^ central memory (CM) γδ T cells with low effector function and strong proliferative potential. Still, upon antigenic stimulation, CM γδ T cells can further mature to CD45RA^−^ CCR7^−^ CD27^+^/^−^ CD28^+^/^−^ effector memory (EM) γδ T cells, producing pro-inflammatory cytokines (IFN-γ, TNF-α) and cytolytic protein (granzymes, perforin). EM γδ T cells finally mature to CD45RA^+^ CCR7^−^ CD27^−^ CD28^−^ re-expressing CD45RA terminally differentiated effector memory (TEMRA) γδ T cells with low proliferative potential and strong cytotoxic function [30,31,32]. While losing the expression to markers associated with immature γδ T cells profiles (e.g., IL7R), the latter express CD16, being able to mediate antibody-dependent cell cytotoxicity (ADCC), and can express CD57, a canonical marker of cellular aging and replicative senescence, also associated with impaired cytotoxic functionality.

Contrasting from their counterpart αβ T cells, γδ T cells display a major histocompatibility complex (MHC)-unrestricted antigen presentation and TCR activation, driving their maturation and the expression of natural killer cells (NK) associated cytotoxic receptors. Indeed, γδ T cells also differ from αβ T cells by expressing natural killer cell receptors such as the NKG2 receptors family. Hence, γδ T cells carry hallmarks of innate as well as adaptive immune responses [33]. In breast cancers, tumor-infiltrating γδ T cells represent up to 15% of T cells, and their prognostic value remains controversial [28,34,35], presumably due to functional divergences of those heterogeneous populations. γδ T cell subpopulation nomenclature depends on the diversity of Vγ and/or Vδ chain used within the TCR and often shows tissue-specific abundance [29,36]. Investigations in breast cancer have mainly focused on Vδ1^+^ γδ T cells since they are the predominant subtype of γδ T cells in breast tissues. Yet Vδ1^+^ γδ T cell functions remain controversial, either affiliated with a Th1 or regulatory polarization [37,38,39]. Oppositely, Vδ2^+^ γδ T cells are a potent pro-inflammatory mediator and cytotoxic effectors towards breast tumor cells and have been observed in direct contact with the latter in breast tumors [40,41,42]. While being weakly represented in breast cancers, Vδ2^+^ γδ T cells are the major subtype of γδ T cells in the peripheral blood [35]. Of note, preclinical models of breast cancers, as well as clinical trials, rationally support the potential clinical benefits from the therapeutic targeting of Vδ2^+^ γδ T cells [43,44,45]. However, more information regarding Vδ2^+^ γδ T cells phenotypic alterations in human breast cancers and their impact on disease progression is needed.

To this end, we used mass cytometry to quantify up to 130 immune variables from the peripheral blood of untreated breast cancer patients. We evidenced peripheral Vδ2^+^ γδ T cells phenotypic alteration detectable at diagnosis of early-stage breast cancer. Notably, peripheral PD-1^+^ or CD57^+^ EMRA Vδ2^+^ γδ T cells are associated with the pathological involvement of tumor-draining axillary lymph nodes.

## 2. Results

### 2.1. Vδ2^+^ γδ T Cell Phenotypic Alterations Are Assessable from the Peripheral Blood of Untreated Breast Cancer Patients

A total of 122 immune variables were quantified from peripheral blood mononuclear cells (PBMCs) of 13 newly diagnosed BC patients and four healthy volunteers (HV) using two mass cytometry panels (Supplementary Materials Table S1). An immune variable corresponds to the frequency of cells displaying positive (co-)expression of targeted markers in our mass cytometry panels. These variables were included in a between-group analysis (BGA) to constitute a composite immune signature discriminating the two groups (BC versus HV).

**Figure 1:**
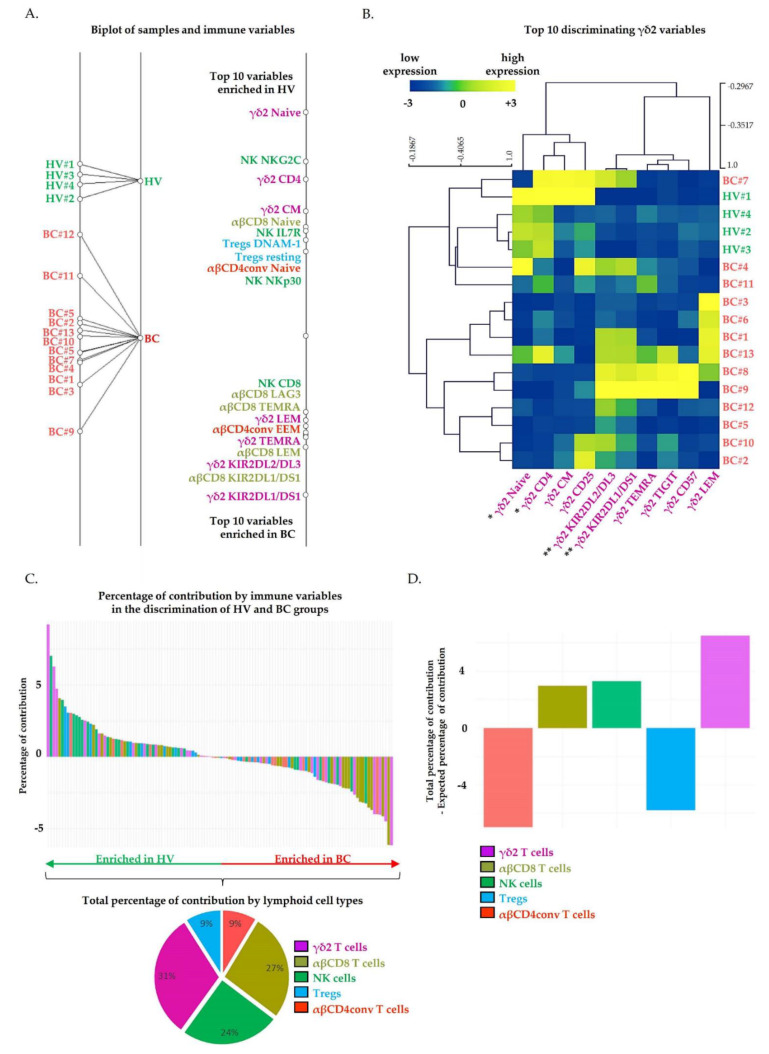
Circulating Vδ2^+^ γδ T cells’ phenotypic alterations contribute markedly to the discrimination of healthy volunteers and breast cancer patients. The results of a between-group analysis (BGA) using 122 circulating immune variables are shown. (**A**) The left axis displays projection of all samples. Samples’ origins annotated in greed correspond to healthy volunteers’ peripheral blood mononuclear cells (PBMC HV, n = 4), and origins annotated in red correspond to breast cancer patients’ peripheral blood mononuclear cells (PBMC BC, n = 13). Each samples’ origin is linked to its own group (black trait). The distances between samples’ origins provide information regarding the degree to which samples were separable. Immune variables are plotted on the right axis. The top 10 discriminating immune variables enriched in healthy volunteers (HV) are shown at the top of the axis; the top 10 discriminating immune variables enriched in BC patients are shown at the bottom of the axis. The discriminating power of each variable is represented by their relative distances to their origin. (**B**) Heatmap visualization of samples’ hierarchical clustering (Pearson’s correlation) based on the normalized top 10 Vδ2 variables. Data were analyzed using Wilcoxon–Mann–Whitney test; *, *p* < 0.05; **, *p* < 0.01. (**C**) The waterfall plot displays the percentage of contribution of each variable to the discrimination of BC from HV groups. Positive percentages of contribution are associated with the HV group. Negative percentages of contribution are associated with the BC group. Pie charts represent the percentage of contribution of immune variables that contribute to group discrimination by lymphoid cell type. (**D**) The bar chart whose Y-axis displays the difference between the total contribution and the expected contribution to each circulating lymphoid population to the discrimination of BC from HV groups.

Because there are only two groups, BGA output is a single dimension discriminating axis, where each sample is positioned according to the coexpression of the 122 variables used as input (Figure 1A, left part). The distance between the origins of the groups provides information on the degree to which groups were separable. The distances between the origins of the samples provide information on the degree to which samples were separable. Neither the origins of the HV and BC samples nor the origins of HV and BC groups overlapped. Thus, the composite immune signature accurately discriminated against the HV and BC samples. Interestingly, BC origins were disparate, highlighting the heterogeneity of the composite immune signature between BC patients.

The top 20 discriminating variables were projected on the single dimension discriminating axis (Figure 1A, right part). For each variable, the distance from the origin represents its relative contribution in the separation of BC from HV samples.

In the group of HV, we detected an increased frequency of NK cells expressing NK triggering receptors (NKp30^+^, NKG2C^+^). Still, in the HV group, T cell populations displayed poorly differentiated and non-activated profiles. Indeed, the frequency of Naïve (CD45RA^+^ CCR7^+^ CD27^+^ CD28^+^) Vδ2^+^ γδ T cells, Naïve αβCD8^+^ T cells, Naïve αβCD4^+^ conventional T cells, central memory (CM, CD45RA^−^ CCR7^+^) Vδ2^+^ γδ T cells, resting (CD45RA^+^ CCR7^+^ CTLA-4^low^ ICOS^low^) Tregs and Tregs expressing a marker associated with a disrupted immunosuppressive activity (DNAM1^+^), were increased in HV samples compared to BC samples. On the opposite, T cells displaying a differentiated and polarized phenotype were associated with the BC group. Indeed, the frequency of effector memory T cells re-expressing CD45RA (TEMRA, CD45RA^+^ CCR7^−^ CD27^−^ CD28^−^) Vδ2^+^ γδ T cells, TEMRA αβCD8^+^, late effector memory (LEM, CD45RA^−^ CCR7^−^ CD27^−^ CD28^−^) αβCD8^+^ T cells and early effector memory (EEM, CD45RA^−^ CCR7^−^ CD27^+^ CD28^+^) αβCD4^+^ conventional T cells were increased in BC samples compared to HV samples. Additionally, an increased frequency of highly cytotoxic (CD8^+^) NK cells was also detected in BC samples. Importantly, frequencies of Vδ2^+^ γδ T, αβCD8^+^ T and NK cells expressing inhibitory receptors (KIR2DL1/DS1^+^, KIR2DL2/DL3^+^, LAG3^+^) were also increased in BC samples.

The variables that are the most discriminating between HV and BC groups are far from the origin in both directions. These variables were mostly related to Vδ2^+^ γδ T cell, with six of the top 10 discriminating variables expressed by Vδ2^+^ γδ T cells. The dot plots displaying the top 20 discriminating immune variables in BC samples versus HV samples are provided in Supplementary Figure S1A,B. Hierarchical clustering based on the top 10 discriminating immune variables allows separating BC from HV samples (Supplementary Figure S2). Interestingly, hierarchical clustering only based on the top 10 discriminating Vδ2^+^ γδ T cells variables also discriminated both groups, which indicates that the phenotypic alterations of Vδ2^+^ γδ T cells are markedly represented in BC samples (Figure 1B).

Next, we determined the percentage of contribution of the 122 variables to the discrimination of HV and BC groups, either individually (Figure 1C, upper part) or aggregated by lymphoid cell type (Figure 1C, lower part). The percentage of contribution by the 122 variables individually are provided in Supplementary Table S2. We identified the top 10 variables enriched in HV as, respectively carrying 9.2% (Vδ2^+^ γδ Naive), 7% (NK NKG2C^+^), 6.3% (Vδ2^+^ γδ CD4^+^), 4.7% (Vδ2^+^ γδ CM), 4.1% (αβCD8^+^ Naive), 4% (NK IL7R^+^), 3.5% (Tregs DNAM1^+^), 3.1%(Tregs resting) and 3% (αβCD4^+^ conventional Naive, NK Nkp30^+^) of contribution to the discrimination of groups (Figure 1C, waterfall plots, green arrow enriched in HV). We identified the top 10 variables enriched in BC as carrying, respectively 6.2% (Vδ2^+^ γδ KIR2DL1/DS1^+^), 6.1% (αβCD8^+^ KIR2DL1/DS1^+^), 4.5% (Vδ2^+^ γδ KIR2DL2/DL3^+^), 4.2% (αβCD8^+^ LEM), 4% (Vδ2^+^ γδ TEMRA, αβCD4^+^ conventional EEM, Vδ2^+^ γδ LEM), 3.7% (αβCD8^+^ TEMRA), 3.5% (αβCD8^+^ LAG3^+^) and 3.2% (NK CD8^+^) of contribution to the discrimination of groups (Figure 1C, waterfall plots, red arrow enriched in BC). By adding the percentage of contribution of each Vδ2^+^ γδ variables (waterfall plots Figure 1C upper part, mauve bar), we evidenced a total percentage of contribution of 31% by Vδ2^+^ γδ T cells variables to the discrimination of groups. We repeated this using the variables expressed by others lymphoid cell types and showed a total percentage of contribution to the discrimination of groups of 27% by αβCD8^+^ T cell variables, 24% by NK cell variables, 9% by Tregs variables, and 9% by αβCD4^+^ T cell variables.

From the 122 immune variables used as input for BGA, a total of 30, 29, 26, 19, 18 variables were, respectively quantified on Vδ2^+^ γδ T, αβCD8^+^ T, NK, αβCD4^+^ conventional T cells and Tregs. That is, by considering each variable as equally contributing to the discrimination of HV and BC groups, Vδ2^+^ γδ T, αβCD8^+^ T, NK, αβCD4^+^ conventional T cells and Tregs, respectively carry an expected percentage of contribution of 25%, 24%, 21%, 16% and 15% to the discrimination of groups. Using BGA, we previously identified a total percentage of contribution of 31% for Vδ2^+^ γδ T cells variables, 27% for αβCD8^+^ T variables, 24% for NK cell variables, 9% for Tregs variables, and 9% αβCD4^+^ T cells variables. By subtracting the expected contribution to the total contribution, for each lymphoid cell type individually, we identified that Vδ2^+^ γδ T cells variables contributed 6% more than expected to the discrimination of groups, versus 3% for αβCD8^+^ T and NK cells variables (Figure 1D). Of note, αβCD4^+^ T cells and Tregs variables contributed less than expected to the discrimination of groups. Collectively, our results evidenced that Vδ2^+^ γδ T cells phenotypic alterations markedly contributed to discriminate HV and BC groups compared to the other lymphoid cell types.

### 2.2. Circulating Vδ2^+^ γδ T Cells Phenotypic Alterations Are Associated with Pathological Lymph Node Invasion in Breast Cancer Patients

**Figure 2:**
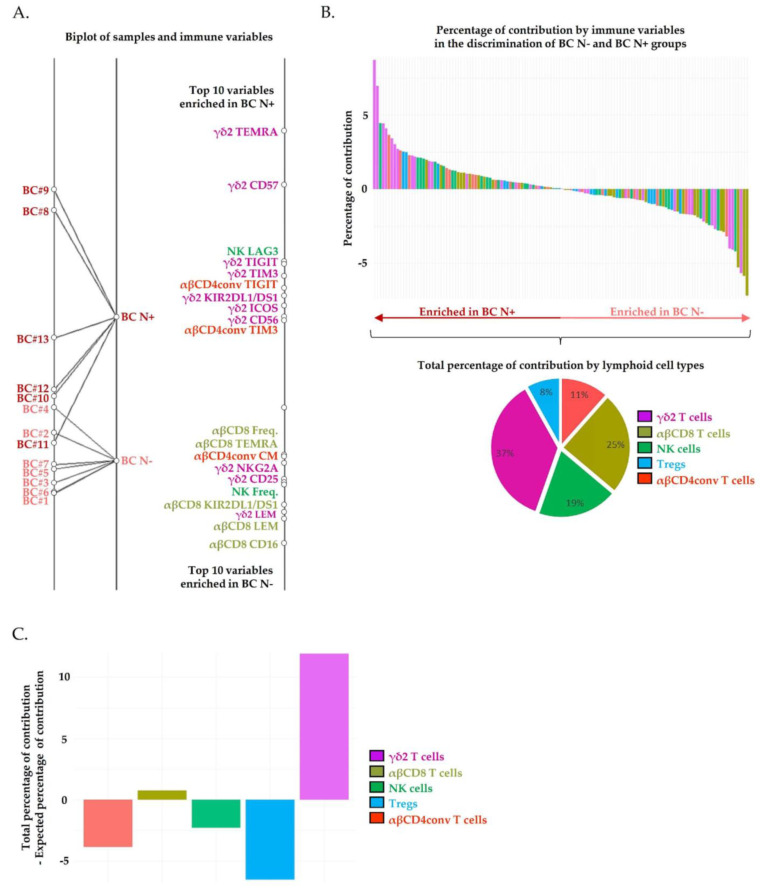
Circulating Vδ2^+^ γδ T cells’ phenotypic alterations are associated with lymph node invasion in breast cancer patients. The results of a BGA using 130 circulating immune variables are shown. (**A**) The left axis displays projection of all patients. Samples origins annotated in dark red correspond to PBMC from breast cancers patients with lymph node invasion (PBMC breast cancer (BC), BC N+, n = 7), and those annotated in light red correspond to PBMC from breast cancers patients without lymph node invasion (PBMC BC, BC N−, n = 6). Each samples’ origin is linked to its own group (black trait). The distances between samples’ origins provide information regarding the degree to which samples were separable. Immune variables are plotted on the right axis. The top 10 discriminating immune variables enriched in patients with lymph node invasion are shown at the top of the axis; the top 10 discriminating immune variables enriched in patients without lymph node invasion are shown at the bottom of the axis. The discriminating power of each variable is represented by their relatives’ distances to their origin. (**B**) The waterfall plot whose Y-axis displays the percentage of contribution of each variable to the discrimination of patients with versus without lymph node invasion. Positive percentages of contribution are associated with the BC N+ group. Negative percentages of contribution are associated with the BC N− group. Pie charts represent the percentage of contribution of immune variables that contribute to group discrimination by lymphoid cell type. (**C**) The bar chart displays the difference between the total contribution and the expected contribution to each circulating lymphoid population to the discrimination of BC N+ and BC N− groups.

The pathological involvement of tumor-draining axillary lymph nodes is the major prognostic factor of early breast cancer. We could quantify 130 immune variables in PBMCs from seven BC patients without tumor-draining lymph nodes invasion (BC N−) and six BC patients harboring invaded tumor-draining lymph nodes (BC N+). Among the 130 immune variables, 32, 31, 20, 19 and 28 variables were, respectively expressed by Vδ2^+^ γδ T cells, αβCD8^+^ T cells, αβCD4^+^ conventional T cells, Tregs and NK cells. These immune variables were included in a BGA to determine a composite immune signature discriminating BC N− and BC N+ groups. BGA shows that BC N− patients were poorly dispersed, while BC N+ patients are characterized by increased inter-patient variability in immunes subpopulation frequencies, resulting in some misclassifications (Figure 2A).

Interestingly, BC N+ patients displayed fully differentiated (TEMRA), activated (ICOS^+^) highly cytotoxic (CD56^+^) Vδ2^+^ γδ T cells profiles. Of note, TEMRA Vδ2^+^ γδ T cells were the unique population harboring a significantly increased frequency in BC N+ patients compared to BC N− patients (Supplementary Figure S3A,B). However, the frequency of Vδ2^+^ γδ T cells expressing inhibitory receptors (TIGIT^+^, TIM3^+^, KIR2DL1/DS1^+^) and a canonical marker of T cell functional replicative senescence (CD57^+^) tends to increase in some patients harboring lymph node invasion, although the difference was not significant. The frequency of NK cells and αβCD4^+^ T cells expressing inhibitory immune checkpoint receptors (respectively LAG3 and TIGIT, TIM3) also tended to increase in some patients harboring lymph node invasion.

The percentages of contribution by the 130 variables individually are provided in supplementary Table S3. We identified the top 10 variables enriched in BC N+ as, respectively carrying 9% (Vδ2^+^ γδ TEMRA), 7% (Vδ2^+^ γδ CD57^+^), 4.5% (NK LAG3^+^, Vδ2^+^ γδ TIGIT^+^), 4.1% (Vδ2^+^ γδ TIM3^+^), 3.6% (αβCD4^+^ conventional TIGIT^+^), 3.4% (Vδ2^+^ γδ KIR2DL1/DS1^+^), 3% (Vδ2^+^ γδ ICOS^+^) and 2.7% (Vδ2^+^ γδ CD56^+^ αβCD4^+^ conventional TIGIT^+^) of contribution to the discrimination of groups (Figure 2B, waterfall plots, dark red arrow enriched in BC N+). We identified the top 10 variables enriched in BC N− as carrying, respectively 7% (Vδ2^+^ γδ KIR2DL1/DS1^+^), 6% (αβCD8^+^ KIR2DL1/DS1^+^), 5% (Vδ2^+^ γδ KIR2DL2/DL3^+^), 4% (αβCD8^+^ LEM) and 3% (Vδ2^+^ γδ TEMRA, αβCD4^+^ conventional EEM, Vδ2^+^ γδ LEM), 3.7% (αβCD8^+^ TEMRA), 3.5% (αβCD8^+^ LAG3^+^) of contribution to the discrimination of groups (Figure 2B, waterfall plots, light red arrow enriched in BC N−).

From the 122 immune variables used as input for BGA, a total of 32, 31, 28, and 19 variables were, respectively quantified on Vδ2^+^ γδ T, αβCD8^+^ T, NK, αβCD4^+^ conventional T cells and Tregs. By considering each variable as equally contributing to the discrimination of HV and BC groups, Vδ2^+^ γδ T, αβCD8^+^ T, NK, αβCD4^+^ conventional T cells and Tregs, respectively carry an expected percentage of contribution of 25%, 24%, 21%, and 15% to the discrimination of groups. Using BGA, we revealed that Vδ2^+^ γδ T cells phenotypic alterations were the main discriminating (Figure 2B). Indeed, the total percentage of contribution by Vδ2^+^ γδ T cells variables was 37%, versus 25% by αβCD8^+^ T cells, 19% by NK cells, 11% by αβCD4^+^ conventional T cells and 8% by Tregs. Overall, by subtracting the expected contribution to the total contribution for each lymphoid cell type individually, we observed that Vδ2^+^ γδ T cells variables contributed 12% more than expected to the discrimination of BC N− and BC N+ groups, far off followed by αβCD8^+^ T cells variables that contributed 1% more than expected (Figure 2C). Of note, NK cells, αβCD4^+^ T cells and Tregs variables contributed less than expected to the discrimination of groups. Collectively, our results evidenced that Vδ2^+^ γδ T cells phenotypic alterations are markedly associated with lymph node invasion compared to the other lymphoid cell types.

### 2.3. High Dimensional Characterization of Vδ2^+^ γδ T Cell Alterations in BC Reveals Association of Senescent/Exhausted TEMRA Phenotypes with Lymph Node Invasion

**Figure 3:**
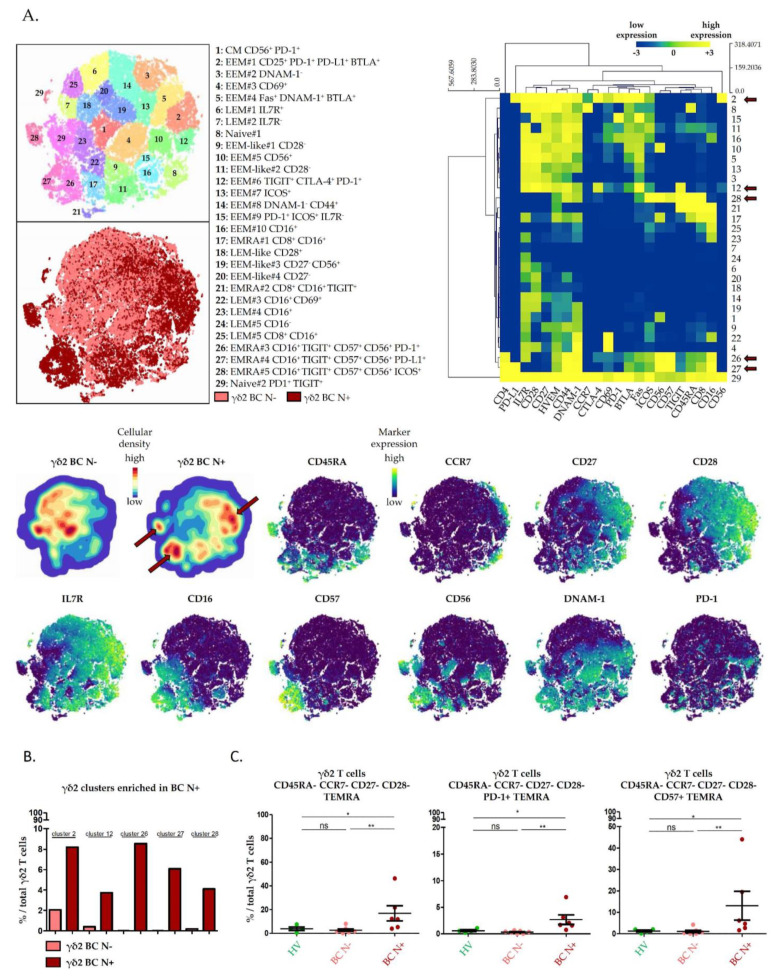
High dimensional characterization of circulating Vδ2^+^ γδ T cells’ phenotypic alterations in breast cancers. Circulating Vδ2^+^ γδ T cell from breast cancer patients with or without lymph node involvement were manually gated, and consensus files were generated for each group of patients with a fixed number of 14,500 Vδ2^+^ γδ T cells. Consensus files were exported in Cytosplore for the *t*-distributed stochastic neighbor embedding dimensionality reduction algorithm (*t*-SNE) analysis. (**A**) Upper left panel: *t*-SNE allows automatic identification of 29 Vδ2^+^ γδ T cell clusters according to the coexpression of 22 markers. Light red cells are from breast cancer patients without lymph node invasion (BC N−). Dark red cells are from breast cancer patients with lymph node invasion (BC N+). Upper right panel: the heatmap summarizes the phenotypic characteristics of Vδ2^+^ γδ T cell subpopulations identified by *t*-SNE. Lower panel: the density of Vδ2+ γδ T cell subpopulations in each group of patients are projected (blue, low cellular density; red, high cellular density). The red arrows in the upper right panel and the lower panel point Vδ2^+^ γδ T cell subpopulations enriched in BC N+. Expressions of markers of Vδ2^+^ γδ T cell differentiation, cytotoxicity, replicative senescence and exhaustion are projected on *t*-SNE maps (blue, low expression; yellow, high expression). (**B**) Bar charts of Vδ2^+^ γδ T cell subpopulations frequencies identified as enriched in patients with lymph node invasion by the *t*-SNE analysis. (**C**) Dot plot of aforementioned Vδ2^+^ γδ T cell subpopulations, identified by manual gating using a minimal number of markers. Data were analyzed using Wilcoxon–Mann–Whitney test, Mean with SEM; *, *p* < 0.05; **, *p* < 0.01; ns, non-significant.

Because the coexpression of Vδ2^+^ γδ T cells variables was poorly considered in previous BGA, we explored the phenotypic diversity of Vδ2^+^ γδ T cells inner BC N− and BC N+ patients liquid biopsies using the *t*-distributed stochastic neighbor embedding dimensionality reduction algorithm (*t*-SNE) (Figure 3A). Twenty-nine Vδ2+ γδ T cells subpopulations were automatically defined, according to the coexpression of 22 variables informative of Vδ2^+^ γδ T cells differentiation stages (CD45RA, CCR7, CD27, CD28, IL7R), cytotoxic potential (CD56, CD16), activation state (CD69, CD44, ICOS), replicative senescence (CD57), inhibitory signaling and tumor-promoting tolerance (PD-1, PD-L1, CTLA-4, BTLA, TIGIT), susceptibility to apoptosis (Fas), tumor cell recognition and costimulatory signaling (DNAM-1) (Appendix A). Echoing the first BGA that associated differentiated immune profiles with the group of BC patients, here most Vδ2^+^ γδ T cells displayed effector memory profiles; 48%, 24% and 17% of Vδ2^+^ γδ T cells clusters were, respectively populations of EEM, LEM and TEMRA.

A total of five Vδ2^+^ γδ T cell clusters that coexpressed inhibitory checkpoint receptors and T cells replicative senescence markers were found to be associated with lymph node involvement (Figure 3A, red arrows, upper right panel heatmap and cellular density plot). Indeed, PD-1^+^ PD-L1^+^ BTLA^+^ EEM (cluster 2), TIGIT^+^ CTLA-4^+^ PD-1^+^ EEM (cluster 12), CD16^+^ CD56^+^ CD57^+^ PD-1^−/+^ TIGIT^+/−^ PD-L1^−/+^ TEMRA (clusters 26, 27, 28) Vδ2^+^ γδ T cells were exclusively found in patients with lymph node involvement (Figure 3B). These variations were confirmed in manual gating: TEMRA Vδ2^+^ γδ T cells were significantly associated with lymph node involvement, confirming the results obtained with the BGA. Importantly, PD-1^+^ and CD57^+^ TEMRA Vδ2^+^ γδ T cells that correspond to clusters 26, 27 and 28, were significantly increased in liquid biopsies of BC N+ patients (Figure 3C).

## 3. Discussion

Recent technologies such as single-cell RNA-sequencing and mass cytometry increased the yield of high-resolution information from small-sized tumor samples. De facto, high dimensional profiling of human breast cancers has already evidenced immune phenotypic alterations paving the way to tumor progression [10,11]. However, to depict a comprehensive portrait of the immune alterations that account for clinical outcomes, a major issue remains tumor specimen accessibility. Indeed, scarce events such as Vδ2^+^ γδ T cells alterations may be prominent at a key moment during BC progression, but underrepresented, if not absent, in small size samples. Here, we managed to bypass the scarcity of those cells in human breast tumor biopsies by phenotyping circulating Vδ2^+^ γδ T cells from liquid biopsies, and we identified Vδ2^+^ γδ T cells phenotypic alterations in the context of human BC.

We first report a composite immune signature that discriminates between healthy and cancerous conditions, suggesting that an antitumor immune response is established and assessable at diagnosis in the peripheral blood. Those results highlight liquid biopsies as potentially relevant samples to monitor global antitumor immune response as reported in previous investigations on αβCD8^+^ T cells, αβCD4^+^ conventional T cells, Tregs, NK cells and monocytes, but not γδ T cells. Indeed, both mRNA and protein expression of major immune regulator molecules, as well as immunosuppressive population frequencies, are increased in the peripheral blood of breast cancer patients [26,46,47]. Moreover, most studies focusing on immune monitoring using liquid biopsies were designed on advanced breast cancers [48,49]. Of note, 93% of BC patients included in our cohort had early-stage tumors. Our BGA, which depicts enriched immune variables related to T cell differentiation and polarization in BC patients, then reveals systemic activation of the host immune system that sets up early during the course of tumor progression. Those results are consistent with recent data conducted at diagnosis, evidencing that nonmetastatic BC induced systemic change on cytokine signaling in circulating lymphocytes [50], as well as increased frequencies of T cells specific to breast tumor-associated antigens in the peripheral blood of BC patients [51].

Given the heterogeneity of the immune composite signature revealed within breast cancer patients’ peripheral blood by the BGA, we carried out a comparison of breast cancer patient’s subgroups defined by clinical parameters related to tumor progression. To determine whether the monitoring of circulating Vδ2^+^ γδ T cells is a relevant track, an evaluation of Vδ2^+^ γδ T cells’ implication in breast cancer pathogenesis and progression should be made in perspective to those of their well-described counterpart lymphoid cells. BGA reveals Vδ2^+^ γδ T cells as the prominent lymphoid cell type whose phenotypic alterations were the most associated with lymph node metastasis. Interestingly, our single cells proteomic profiling of liquid biopsies is consistent with previous bulk transcriptional profiling of breast tumors, evidencing γδ T cells as the lymphoid population carrying the most favorable prognosis in breast cancers [28,52].

Vδ2^+^ γδ T cells cytotoxicity is strongly assigned to CD45RA^+^ CCR7^-^ CD27^-^ CD28^-^ TEMRA phenotypes [53]. BGA revealed that frequencies of cytotoxic TEMRA Vδ2^+^ γδ T cells were significantly increased in patients with pathologically invaded tumor-draining lymph nodes. Moreover, the *t*-SNE analysis revealed that cytotoxic TEMRA Vδ2^+^ γδ T cells that are enriched in BC N+ patients also display the positive expression of CD16 and CD56 proteins, which has been associated with enhanced Th1 function and tumor-induced degranulation capacity by Vδ2^+^ γδ T cells, comforting their highly cytotoxic and pro-inflammatory potential towards tumor cells [54,55]. However, these TEMRA displayed features of senescence and exhaustion, with a high expression of PD1, a key inhibitory receptor in T cells tolerance induction [56,57], as well as CD57, a canonical marker of replicative Vδ2^+^ γδ T cells senescence associated with impaired cytotoxic function toward tumor cells and diminished TNF-α/IFN-γ release [58,59].

Overall, our results highlight circulating Vδ2^+^ γδ T cells phenotypic alteration at diagnosis of breast cancers, with increased frequencies of PD-1^+^ and CD57^+^ TEMRA Vδ2^+^ γδ T cells associating with the pathological involvement of tumor-draining axillary lymph nodes. Whether increased frequencies of exhausted/senescent TEMRA Vδ2^+^ γδ T cells reflect altered migratory potential inwards breast tumor bed and/or tumor-infiltrating Vδ2^+^ γδ T cells, phenotypic alteration remains to be determined and confirmed in a larger cohort, to assess the interest of using liquid biopsies as a source of a surrogate marker for the monitoring of immunotherapeutic clinical trials targeting Vδ2^+^ γδ T cells.

## 4. Materials and Methods

### 4.1. Study Participants

All patients’ peripheral blood specimens were obtained through the prospective biobank “BC-BIO” IPC protocol (NCT01521676) approved by the institutional review board (Comité d’Orientation Stratégique (COS), Marseille, France) of the Paoli-Calmettes Institute. Written informed consent was obtained from all patients in concordance with the Declaration of Helsinki. Age-matched healthy subjects were recruited from the Etablissement Français du Sang (EFS Alpes-Méditerranée).

### 4.2. Clinical Samples

Breast cancer patients treated at the Institut Paoli-Calmettes were prospectively enrolled between February 2018 and June 2018. Fresh EDTA-anticoagulated blood samples were obtained from patients at diagnosis (n = 13) and healthy volunteers (N = 4). Blood samples were processed extemporaneously. After analysis of morphological tumor characteristics by pathologists, patients were classified as summarized in Supplementary Table S4.

### 4.3. Mass Cytometry Staining and Data Acquisition

PBMCs were obtained using Ficoll-Paque density-gradient centrifugation. Cells were consecutively centrifuged and incubated with cisplatin 0.1 M to stain dead cells. Aspecific epitope binding was blocked with 0.5 mg/mL human Fc Block (BD Biosciences, San Jose, CA, USA). The two mass cytometry panels used are provided in Supplementary Table S1. Two million PBMCs were stained for 1 h at 4 °C with the mix of extracellular antibodies then 1 h with secondary antibodies. After centrifugation, cells were washed and permeabilized with the Foxp3 staining buffer set (eBioscience, San Diego, CA, USA) for 30 min at 4 °C. Intracellular aspecific epitopes were blocked with 0.5 mg/mL human Fc Block for 10 min at 4 °C before incubation with the mix of intracellular antibodies for 1 h at 4 °C in Foxp3 staining buffer. Cells were then washed and labeled overnight with 125 nM iridium intercalator (Fluidigm, South San Francisco, CA, USA) in Cytofix (BD Biosciences). Finally, cell pellets were resuspended in Milli-Q water (Merck Millipore, Burlington, MA, USA) containing 10% EQ four element calibration beads (Fluidigm) and filtered through a 35 μm membrane before acquisition on a mass cytometer (Helios^®^ instrument, Fluidigm), at an acquisition rate of approximately 500 events per second. Following the manufacturer’s instructions, settings were on default.

### 4.4. Data Processing and Analysis

#### 4.4.1. Between-Group Analysis (BGA)

TCRγδ2^+^ T cells, αβCD8^+^ T cells, αβCD4^+^ conventional T cells, Tregs and NK cells were manually gated according to the gating strategy displayed in Supplementary Figure S4 using FlowJo V10.6.2. After exclusions of variables with mean expression < 3% to avoid any assessment of background noises, the between-group analysis (BGA) [60] was performed using RStudio V1.3.1093, R-4.0.3 (made4 package). R scripts performing BGA are provided in File S1. BGA analysis outputs the contribution of each immune variable as well as the relative contribution of each immune cell type to the discrimination of the compared groups. These data are provided in Supplementary Tables S2 and S3. Hierarchical clustering (Pearson’s correlation) and heatmap visualization were generated using MeV V4.9.0 [61].

#### 4.4.2. *t*-Distributed Stochastic Neighbor Embedding Analysis (*t*-SNE)

Manually gated Vδ2^+^ γδ T cells were exported using FlowJo V10.6.2. Prior to *t*-SNE analyses using Cytosplore V2.2.1 [62], consensus files were generated for each group of patients by concatenation using FlowJo V10.6.2. Consensus files were imported in Cytosplore using an arcsine transformation with a cofactor of 5 and downsampled to a total number of 14,500 Vδ2^+^ γδ T cells for each file. The *t*-SNE analysis was carried out with the default setting (perplexity of 30 and 1000 iterations), and Vδ2^+^ γδ T cell subpopulations were automatically defined by cell density gradient. The results of the *t*-SNE analysis for each panel are provided in Appendix A. Hierarchical clustering (Pearson’s correlation), and heatmap visualization were generated using MeV V4.9.0.

### 4.5. Statistical Analysis

Statistical analyses were generated using GraphPad Prism V5.00. Data are expressed as mean ± standard error of the mean (SEM). Statistical significance between two groups was calculated using the nonparametric Mann–Whitney test. A *p*-value < 0.05 was considered as significant.

## 5. Conclusions

Circulating Vδ2^+^ γδ T cells, phenotypic alterations were evidenced in newly diagnosed breast cancer patients. PD-1^+^ or CD57^+^ EMRA Vδ2^+^ γδ T cells associated with lymph node invasion. These findings evidenced in the context of an exploratory study require confirmation in a larger cohort. However, they open new perspectives for the development of Vδ2^+^ γδ T cell immunotherapy in breast cancer.

## Figures and Tables

**Figure A1:**
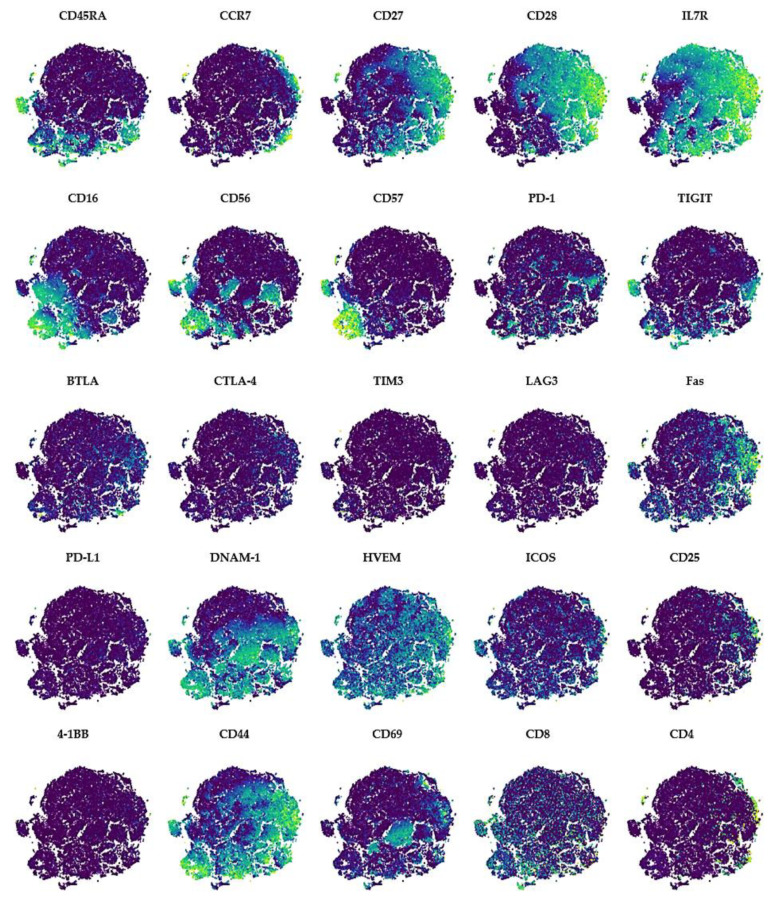
High dimensional characterization of circulating Vδ2^+^ γδ T cells’ phenotypic alterations in breast cancers. The expressions of markers of Vδ2^+^ γδ T cell differentiation (CD45RA, CCR7, CD27, CD28, IL7R), cytotoxicity (CD16, CD56), co-stimulation (4-1BB, CD25, HVEM, ICOS), activation (CD44, CD69), susceptibility to apoptosis (Fas), tumor cell recognition (DNAM-1), replicative senescence (CD57) and exhaustion (PD-1, TIGIT, BTLA, CTLA-4, TIM3, LAG3, HVEM) are projected on *t*-SNE maps (blue, low expression; yellow, high expression).

## Data Availability

The data presented in this study are available on request from the corresponding author.

## Supplementary Materials

The following are available online at https://www.mdpi.com/2072-6694/13/3/441/s1, Figure S1: Frequency comparison of the top 20 circulating immune variables discriminating HV and BC patients’ groups, Figure S2: Heatmap of the top 10 immune variables discriminating HV and BC patients’ groups, Figure S3: Frequency comparison of the top 20 circulating immune variables discriminating BC N− and BC N+ groups. Figure S4: Manual gating strategy of PBMC populations and T cells subpopulations of a representative sample, Table S1: Mass cytometry panels, Table S2: BGA HV vs. BC samples, Table S3: BGA BC N− vs. BC N+ samples, Table S4: Patients’ baseline clinicopathological characteristics, File S1: BGA R scripts.
