# Supplementary material for: Quantification of Immune Variables from Liquid Biopsy in Breast Cancer Patients Links Vδ2+ γδ T Cell Alterations with Lymph Node Invasion
Source: Cancers (Basel). 2021 Jan 25;13(3):441. doi: 10.3390/cancers13030441 (PMC7865589; doi:10.3390/cancers13030441)
Supplement: Supplementary file 1 [file cancers-13-00441-s001.zip › cancers-1039857-supplementary.pdf]

cancers

MDPI

Quantification of Immune Variables from Liquid Biopsy in Breast Cancer Patients Links Vδ2⁺ γδ T Cell Alterations with Lymph Node Invasion

Stéphane Fattori, Laurent Gorvel, Samuel Granjeaud, Philippe Rochigneux, Marie-Sarah Rouvière, Amira Ben Amara, Nicolas Boucherit, Magali Paul, Marie Mélanie Dauplat, Jeanne Thomassin-Piana, Maria Paciencia-Gros, Morgan Avenin, Julien Barrou, Gilles Houvenaeghel, Emmanuelle Charafe-Jauffret, Eric Lambaudie, François Bertucci, Anthony Goncalves, Carole Tarpin, Jacques A. Nunès, Raynier Devillier, Jihane Pakradouni, Anne-Sophie Chretien and Daniel Olive

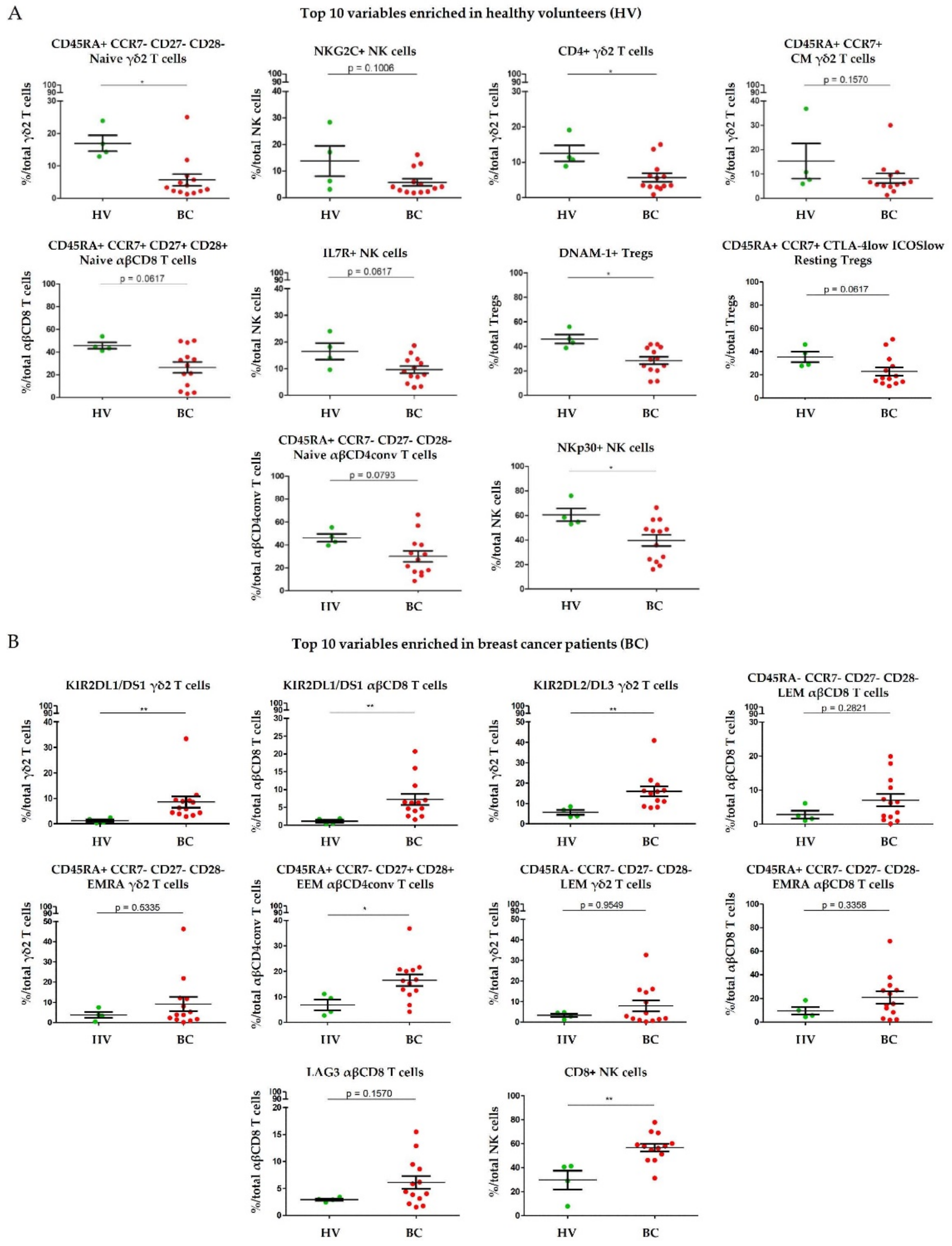

Figure S1. Frequency comparison of the top 20 circulating immune variables discriminating HV and BC patients' groups. Data were analyzed using Wilcoxon–Mann–Whitney test, Mean with SEM ; *, P<0.05 ; **, P<0.01.

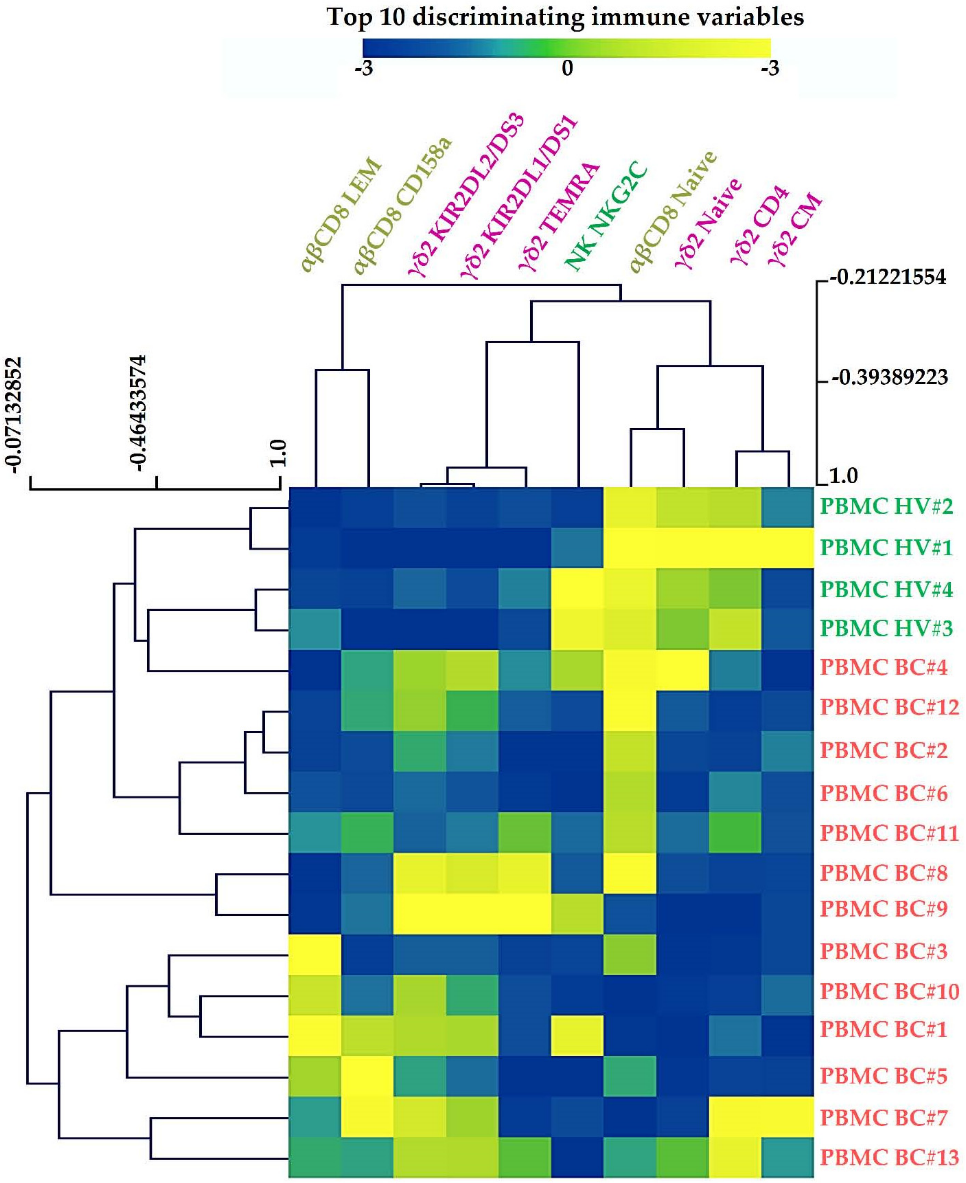

Figure S2. Heatmap visualization of samples' hierarchical clustering (Pearson's correlation) based on the normalized top 10 discriminating immune variables.

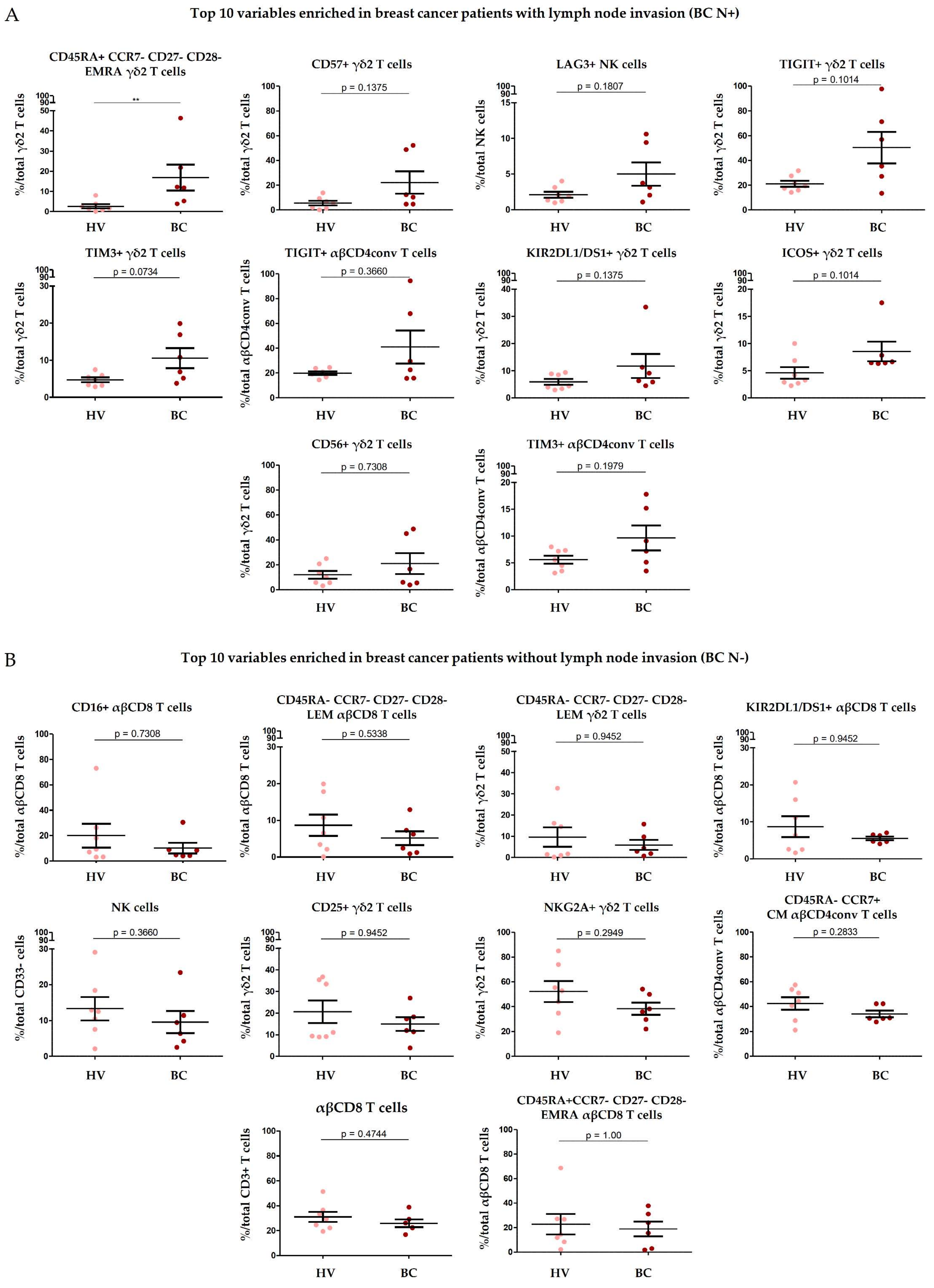

Figure S3. Frequency comparison of the top 20 circulating immune variables discriminating BC N- and BC N+ groups. Data were analyzed using Wilcoxon–Mann–Whitney test, Mean with SEM ; *, $P<0.05$; **, $P<0.01$.

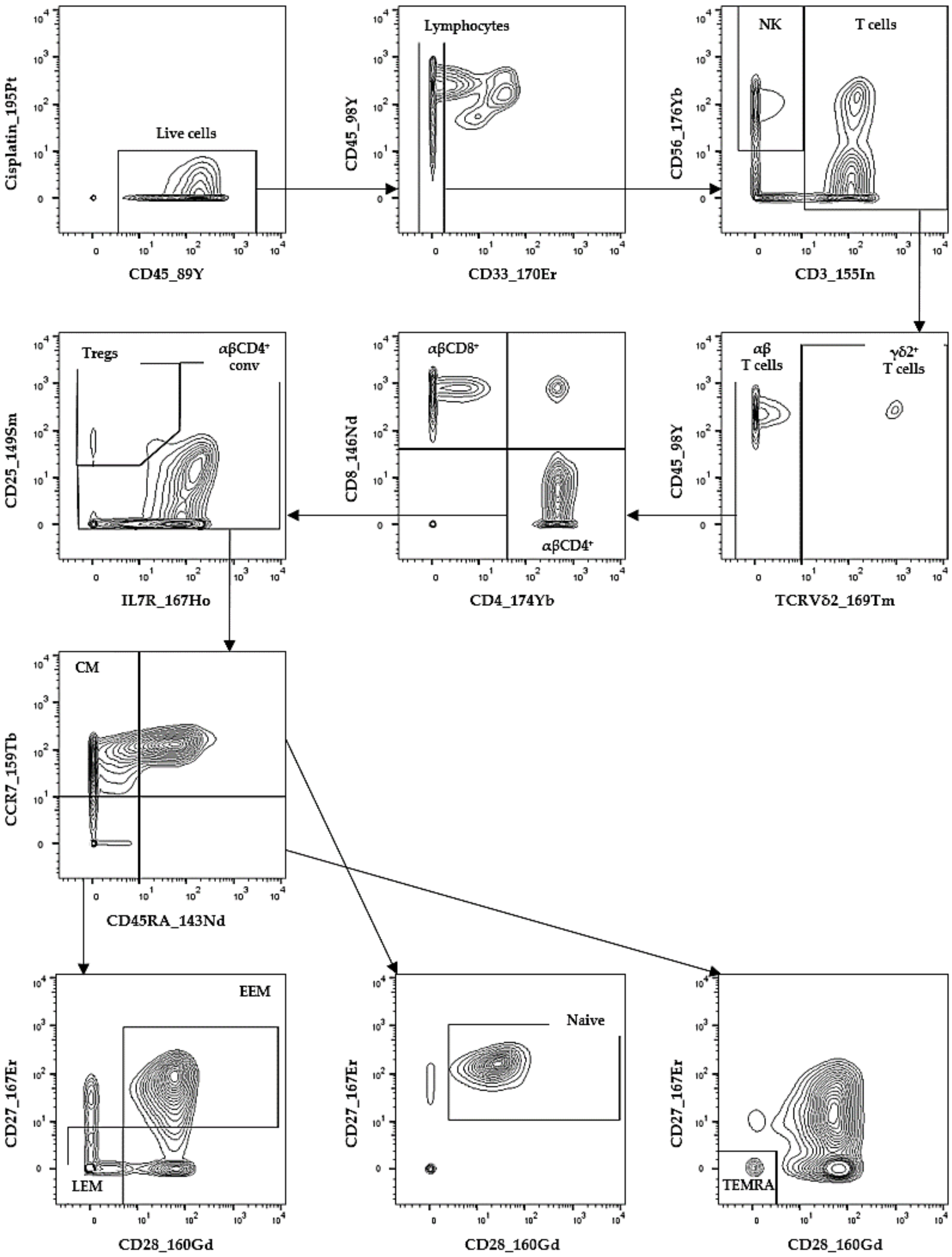

Figure S4. Manual gating strategy of PBMC populations and T cells subpopulations of a representative sample.

Table S1. Mass cytometry panels.

Panel_1

Extracellular staining			
CD45	89	Y	Fluidigm
CD3	115	In	Purified : Ozyme/metal labeling kit: Fluidigm
CD45RA	143	Nd	Fluidigm
HVEM	144	Nd	Fluidigm
CD8a	146	Nd	Fluidigm
CD25	149	Sm	Fluidigm
ICOS	151	Eu	Fluidigm
TIM-3	153	Eu	Fluidigm
TIGIT	154	Sm	Fluidigm

PD-1	155	Gd	Fluidigm
PD-L1	156	Gd	Fluidigm
4-1BB	158	Gd	Fluidigm
CCR7	159	Tb	Fluidigm
CD28	160	Gd	Fluidigm
BTLA	163	Dy	Fluidigm
Fas	164	Dy	Fluidigm
IL-7R	165	Ho	Fluidigm
CD44	166	Er	Fluidigm
CD27	167	Er	Fluidigm
CD69	168	Er	Fluidigm
TCRVd2	169	Tm	Purified : Ozyme/metal labeling kit: Fluidigm
DNAM-1	171	Yb	Fluidigm
CD57	172	Yb	Fluidigm
CD4	174	Yb	Fluidigm
LAG3	175	Lu	Fluidigm
CD56	176	Yb	Fluidigm
CD16	209	Bi	Fluidigm
CD33		biotin	Ozyme
Secondary antibody			
Anti-biotin	170	Er	Fluidigm
Intracellular Staining			
CTLA-4	161	Dy	Fluidigm
		**Panel_2**	
Extracellular staining			
CD45	89	Y	Fluidigm
CD3	115	In	Purified : Ozyme/metal labeling kit: Fluidigm
TCRVd2	141	Pr	Purified : Ozyme/metal labeling kit: Fluidigm
CD19	142	Nd	Fluidigm
CD45RA	143	Nd	Fluidigm
CD4	145	Nd	Fluidigm
CD8	146	Nd	Fluidigm
NKG2C	152	Sm	Fluidigm
KIR2DL2/DL3	154	Sm	Purified: Ozyme/metal labeling kit: Fluidigm
CD27	155	Gd	Fluidigm
NKG2D	160	Gd	Fluidigm
NKp46	162	Dy	Fluidigm
DNAM-1	164	Dy	Fluidigm
NKG2A	165	Ho	Fluidigm
CD96	166	Er	Fluidigm
KIR2DL1/DS1	168	Er	Purified : Ozyme/metal labeling kit: Fluidigm
NKp30	169	Tm	Fluidigm
CD57	172	Yb	Fluidigm
CD56	176	Yb	Fluidigm
CD33		biotin	Ozyme
Secondary antibody			
Anti-biotin	170	Er	Fluidigm

Table S4. Patients' baseline clinicopathological characteristics.

**N patients**	**13**
Sex, n (%)	Women, 13 (100%)
**Age, median (range)**	63 (35–81) years
**Tumor type**	
ductal, n (%)	11 (85)
lobular, n (%)	2 (15)
**Clinical tumor stage**	
pT1a [1], n (%)	1 (8)
pT1b [2], n (%)	1 (8)
pT1c [3], n (%)	7 (54)
pT2 [4], n (%)	4 (30)
pT3 [5], n (%)	0 (0)
pT4 [6], n (%)	0 (0)
**Histological grade**	
G I, n (%)	3 (23)
G II, n (%)	6 (46)
G III, n (%)	4 (31)
**Lymph node invasion**	
N+	6 (46)
N−	7 (54)
**ER/PR/HER2 status (IHC)**	
H+, n (%)	10 (77)
H−, n (%)	3 (23)
HER2+, n (%)	6 (46)
HER2−, n (%)	7 (54)
Triple negative, n (%)	3 (21)
Ki-67 0–19% of positive cells, n (%)	6 (46)
Ki-67 > 20% of positive cells, n (%)	7 (54)

[1] tumor 0.1–0.5 cm; [2] tumor 0.5–1.0 cm; [3] tumor 1.0–2.0 cm; [4] tumor 2.0–5.0 cm; [5] tumor > 5 cm; [6] invasion to chest/skin/inflammatory BC; N+, positive lymphatic nodes; N-, negative lymphatic nodes; ER, estrogen receptors; PR, progesterone receptors; HER2, human epidermal growth factor receptor 2; H, hormone receptors; H+, positive for hormonal receptors; HER-, negative for hormonal receptors; HER2+, positive for HER2; HER2-, negative for HER2; triple negative, negative for H and HER2.
